# An Analytical Method for Assessing Stage-Specific Drug Activity in *Plasmodium vivax* Malaria: Implications for *Ex Vivo* Drug Susceptibility Testing

**DOI:** 10.1371/journal.pntd.0001772

**Published:** 2012-08-07

**Authors:** Douglas H. Kerlin, Kane Boyce, Jutta Marfurt, Julie A. Simpson, Enny Kenangalem, Qin Cheng, Ric N. Price, Michelle L. Gatton

**Affiliations:** 1 Malaria Drug Resistance and Chemotherapy Laboratory, Queensland Institute of Medical Research, Brisbane, Australia; 2 Menzies School of Health Research and Charles Darwin University, Darwin, Australia; 3 Centre for Molecular, Environmental, Genetic and Analytic Epidemiology, University of Melbourne, Melbourne, Australia; 4 Papuan Health and Community Development Foundation (PHCDF), Timika, Papua, Indonesia; 5 District Health Authority and the Menzies School of Health Research-National Institute of Health Research and Development, Timika, Papua, Indonesia; 6 District Ministry of Health, Timika, Papua, Indonesia; 7 Department of Drug Resistance and Diagnostics, Australian Army Malaria Institute, Brisbane, Australia; 8 Centre for Tropical Medicine, Nuffield Department of Clinical Medicine, University of Oxford, Oxford, United Kingdom; Eijkman-Oxford Clinical Research Unit, Indonesia

## Abstract

The emergence of highly chloroquine (CQ) resistant *P. vivax* in Southeast Asia has created an urgent need for an improved understanding of the mechanisms of drug resistance in these parasites, the development of robust tools for defining the spread of resistance, and the discovery of new antimalarial agents. The *ex vivo* Schizont Maturation Test (SMT), originally developed for the study of *P. falciparum*, has been modified for *P. vivax*. We retrospectively analysed the results from 760 parasite isolates assessed by the modified SMT to investigate the relationship between parasite growth dynamics and parasite susceptibility to antimalarial drugs. Previous observations of the stage-specific activity of CQ against *P. vivax* were confirmed, and shown to have profound consequences for interpretation of the assay. Using a nonlinear model we show increased duration of the assay and a higher proportion of ring stages in the initial blood sample were associated with decreased effective concentration (EC_50_) values of CQ, and identify a threshold where these associations no longer hold. Thus, starting composition of parasites in the SMT and duration of the assay can have a profound effect on the calculated EC_50_ for CQ. Our findings indicate that EC_50_ values from assays with a duration less than 34 hours do not truly reflect the sensitivity of the parasite to CQ, nor an assay where the proportion of ring stage parasites at the start of the assay does not exceed 66%. Application of this threshold modelling approach suggests that similar issues may occur for susceptibility testing of amodiaquine and mefloquine. The statistical methodology which has been developed also provides a novel means of detecting stage-specific drug activity for new antimalarials.

## Author Summary

The schizont maturation test (SMT) was developed to monitor drug resistance in malaria parasites. The SMT examines differences in the rate of parasite development when exposed to different drug concentrations, providing an estimate of drug efficacy. While the assay is effective when examining resistance in *Plasmodium falciparum*, there are concerns regarding its suitability for testing other malaria species, particularly if the drug only targets particular life-cycle stages of the parasite. Blood samples taken from *Plasmodium vivax* infected individuals exhibit significant heterogeneity in the parasite life-cycle stages present. If a drug targets the early stage parasites, but only late stage parasites are present in the sample, the test will show an erroneously high degree of resistance. In this study, we estimate thresholds which can be used to identify when test results can be considered accurate should the drug being tested only affect specific life stages of the parasites. Chloroquine is used as a case study but the method developed also allows the identification of stage-specific activity in other malarial drugs in *P. vivax*. For field researchers, this threshold modelling approach will allow for increased confidence in the reliability of *P. vivax* resistance results and provides a novel means of detecting stage-specific drug activity for new antimalarials.

## Introduction

Malaria continues to pose a significant threat to human health globally. Currently, as many as 2.6 billion people are at risk of *P. vivax* infection, with an estimated 72–390 million cases per year [1], [2]. Historically, malaria research has focussed on *P. falciparum*, due to its reputation as the most lethal human malaria parasite. Although *P. vivax* is less pathogenic than *P. falciparum*, it still poses a serious public health burden and is being increasingly recognised as a cause of severe and fatal disease, particularly in children and pregnant women [2], [3].

A number of recent publications have highlighted the increasing recognition of the clinical importance of *P. vivax* and the renewed emphasis placed on research into this species [2], [4], [5], [6]. Further, the emergence of highly chloroquine (CQ) resistant *P. vivax* in Southeast Asia (CQ remains a front-line treatment for vivax malaria as it is affordable, well tolerated and safe, and its long half-life ensures protection from early relapses [7], [8]) has created an urgent need for an improved understanding of the mechanisms of drug resistance in these parasites, the development of robust tools for defining the spread of resistance, and the discovery of new antimalarial agents. To glean insights into the development of resistance in *P. vivax*, a modified form of the *ex vivo* Schizont Maturation Test (SMT) has been developed and applied to fresh isolates directly from patients [9], [10], [11], [12].

The central tenant of the SMT is that drug activity on susceptible parasites will completely stop or slow the growth in a dose-dependent manner, with reduced susceptibility being manifest by an ability of parasites to mature to schizont stages in the presence of higher concentrations of drug. The standard assay is conducted for 30 hours, the time required for parasites to reach maturation without drug, and the proportion of schizonts at the conclusion of the assay is used as an indicator of parasite maturation.

The SMT was initially developed for testing drug susceptibility in *P. falciparum* [13], [14], where almost all parasites in the peripheral circulation are at the immature ring stage. However, in infections due to non-falciparum species, trophozoite and schizont stages are commonly present in the peripheral circulation. To accommodate this, a modified SMT has been developed in which the control wells are monitored until the number of schizonts exceeds 40% of parasites prior to harvest (i.e., assays are conducted for variable lengths of time) [15].

For the results of the SMT to be valid, a sample, irrespective of the drug resistance phenotype of the parasites, must have had sufficient exposure to the drug to affect a response. The diversity in parasite life cycle stages in *P. vivax* infections creates a significant confounding factor, particularly since there is an apparent marked variation of drug susceptibility in different erythrocytic life cycle stages. In previous work, it has been demonstrated that the trophozoite stages of *P. vivax* are almost completely resistant to CQ, and continue to mature no matter how high the concentration of drug [15], [16]. It follows that, if a drug only acts on ring stage parasites, but the sample contains a majority of trophozoites and schizonts, the parasite is likely to be erroneously categorised as resistant simply because there were no susceptible life cycle stages present in the assay.

In this study we develop a statistical methodology to identify stage specific drug activity in the SMT. We use CQ against *P. vivax* as a case study and demonstrate that stage specific drug activity has profound consequences for the interpretation of SMT results. We also examine stage-specific drug effects for other commonly used drugs and simulate the growth dynamics of *P. vivax* parasites within the SMT to provide recommendations on how to improve the reliability of SMT results.

## Methods

### Ethics Statement

Ethical approval for the collection of blood samples for drug susceptibility testing was obtained from the ethics committees of the National Institute of Health Research and Development, Ministry of Health (Jakarta, Indonesia), and the Human Research Ethics Committee of NT Department of Health & Families and Menzies School of Health Research (Darwin, Australia). Written informed consent was obtained from adult patients and parents and/or guardians of enrolled children.

### Data collection and *ex vivo* drug susceptibility testing

Blood samples were collected from patients attending outpatient clinics in Timika, Papua Province, Indonesia, as previously described [15]. Only patients infected with a single species of Plasmodia were included in the study; the majority of samples contained *P. vivax* or *P. falciparum*, although a limited number of *P. malariae* and *P. ovale* isolates were also available [17].

SMTs were conducted on these samples following World Health Organisation guidelines for drug susceptibility testing [18], with modifications developed to aid the application of the test to *P. vivax* [15]. Venous blood (5 mL) was collected by venipuncture, and after removal of host white blood cells using a CF11 column, 800 µL of packed infected red blood cells (IRBC) were used for the SMT. Assays conducted for up to 7 drugs: CQ, artesunate, amodiaquine, lumefantrine, mefloquine, piperaquine and pyronaridine [15].

The proportion of parasite life cycle stages (i.e., rings, trophozoites and schizonts, as defined by Russell et al [12], [15]) in each isolate was assessed at 0 hours, 24 hours, and then at non-uniform times until 40% of the parasites in the control well reached mature schizonts. At this point, the assay was terminated, and wells under serial drug concentrations were harvested. The proportion of parasites in each stage of the erythrocytic cycle at time of harvest was reported for each drug concentration, as was the duration of the assay.

### Dose Response Modelling

The estimated drug response (*R*) of each isolate was derived from the ratio between the proportion of schizonts at harvest in the treatment well compared to that in the control well. Only data satisfying the following criteria were included in the dose response modelling:

1. *R*<1.5 for all drug concentrations,
2. *R*>0 for the lowest drug concentrations,
3. *R*<0.5 for at least one drug concentration.

The sigmoid E_max_ dose-response curve (equation 1) was fitted to all available data simultaneously, with mixed-effects modelling used for CQ data.


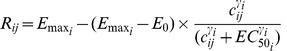
(1)

*R_ij_* represents the drug mediated growth inhibition response (the ratio between the proportion of schizonts at harvest in the treatment well compared to the control well) for the i^th^ isolate at the j^th^ concentration, *c_ij_* represents the drug concentration. *E_max_* and *E_0_* represent the maxima and minima of the dose response curve and *γ* the slope of this curve. The EC_50_ value represents the effective concentration at which 50% of the parasite population exhibit a response to the drug. Inter-isolate variability was included for E_max_, EC_50_ and *γ*. The drug plate batch was also incorporated as a random effect, to control for batch-to-batch variability. Predicted values for EC_50_ for each isolate were calculated from Empirical Bayes estimates.

EC_50_ values for parasite samples collected from April 2004 and May 2007 have previously been reported (Russell 2008). However the methodology used to calculate EC_50_ for CQ differs between the previous report and the current study due to the use of mixed-effects modelling.

### Statistical analysis

For each of the seven drugs tested, linear regression models were used to characterise the relationship between the EC_50_ values derived for each isolate and the following independent variables: assay duration, the proportion of rings at 0 hours, and delay between venepuncture and assay. The EC_50_ values were not normally distributed, therefore ln(EC_50_) was used as the dependent variable.

Where a relationship between one of the independent variables and ln(EC_50_) was found a non-linear threshold model was constructed to determine the threshold at which the association ceased to exist (Equation 2).


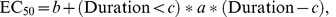
(2)

*a* represents the rate of decline in EC_50_ when the duration of the assay is less than the threshold, *b* the mean EC_50_ values for samples where the duration exceeds the threshold, and *c* the threshold. A threshold model was preferred over other non-linear models due to the greater interpretability of parameters.

A threshold model of the same form was also fitted to define the relationship between the proportion of rings at time 0 and EC_50_. The threshold models were fitted to the data using nonlinear regression.

### Characterising the duration of the erythrocytic cycle

A simulation approach was used to estimate the duration of each stage of the erythrocytic life cycle in both *P. vivax* and *P. falciparum*. Estimates were also made of stage durations using the limited data available for *P. malariae* and *P. ovale* to validate the methodology. Poisson distributions were selected to represent the duration of each stage, as random deviates produced by sampling the distribution are always positive, and the parameters of the distributions allow for biologically meaningful interpretation. We assumed the duration of each stage could be drawn from a probability distribution, and estimate the mean of each Poisson distribution (λ).

One hundred simulations of the growth dynamics within a culture well, each containing 200 parasites, were conducted. It was assumed each parasite began life as a ring, transitioned to a trophozoite, then to a schizont. The length of time spent in each stage was determined by sampling from the relevant probability distribution. At selected time points, the proportion of parasites in each stage was calculated. As parasites completed the schizont stage, they were assumed to die, and subsequently, proportions were calculated based only on the remaining surviving parasites.

A systematic search of the parameter space for the mean duration in each life cycle stage (λ) was conducted to determine the optimal values for each Poisson distribution. The mean model fit from three simulation experiments (totalling 300 simulations) for each combination of parameters was used and the search examined potential parameters in increments of 0.5 hours.

The optimal fit was determined by minimising the sums-of-squares between the simulation results and data on proportion of parasite at each life cycle stage from the control wells, for a subset of the original field samples. The subset of field samples used had 1) 100% rings at 0 hours, 2) data for at least 3 time points, and 3) an assay duration >42 hours. This subset was used to ensure only samples with young ring stage parasites were included in the fitting process. A penalty equivalent to a difference of 20 between data and simulation results was applied for any later time points where the death of the entire simulated parasite population meant that no direct comparison could be made to the data.

All statistical analyses and simulations were conducted using the R statistical computing software package [19].

## Results

### Description of initial samples (at 0 hours)

SMT results for parasites sourced from 784 patients with single-species infections of either *P. vivax* (*n* = 345) or *P. falciparum* (*n* = 439) were analysed; 289 (84%) *P. vivax* and 331 (75%) *P. falciparum* isolates met the inclusion criteria for statistical analysis. Among these, 141 *P. vivax* (49%) and 216 *P. falciparum* (65%) isolates reached the 40% schizont threshold at harvest. The time between venepuncture and start of the assay (the ‘delay’) was significantly correlated with the duration of the assay for isolates of *P. falciparum* (correlation co-efficient (*r*) = −0.211, *p*<0.001), but not *P. vivax* (*r* = −0.065, *p* = 0.226).

There was a significantly higher mean proportion of ring stage parasites in samples at the start of the assay (0 hours) for *P. falciparum* (0.922) compared to *P. vivax* (0.588) (*Wilcoxon rank sum test*, *p*<0.001). 87.5% (189/216) of *P. falciparum* isolates contained 100% ring stage parasites at 0 hours, compared to only 2.8% (4/141) of *P. vivax* isolates.

### Predictors of CQ susceptibility

**Figure 1:**
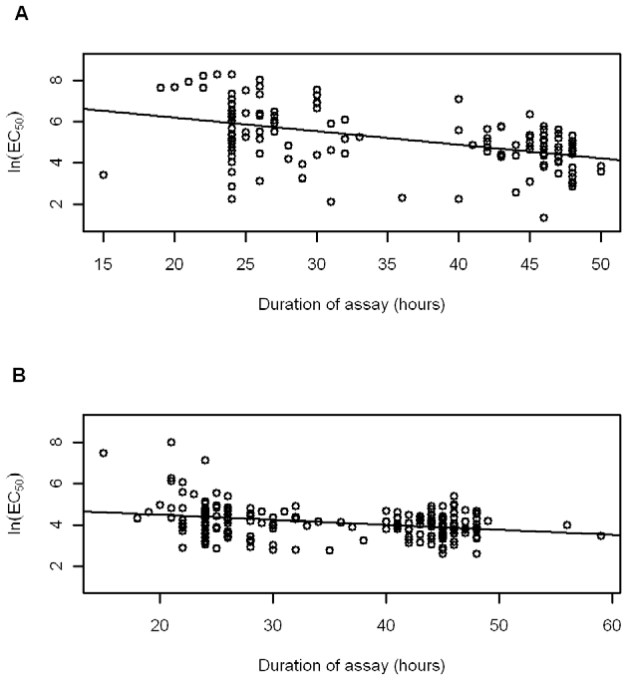
Relationship between assay duration and ln(EC_50_). The figure depicts relationships for (A) *P. vivax* (n = 141) and (B) *P. falciparum* (n = 216).

**Figure 2:**
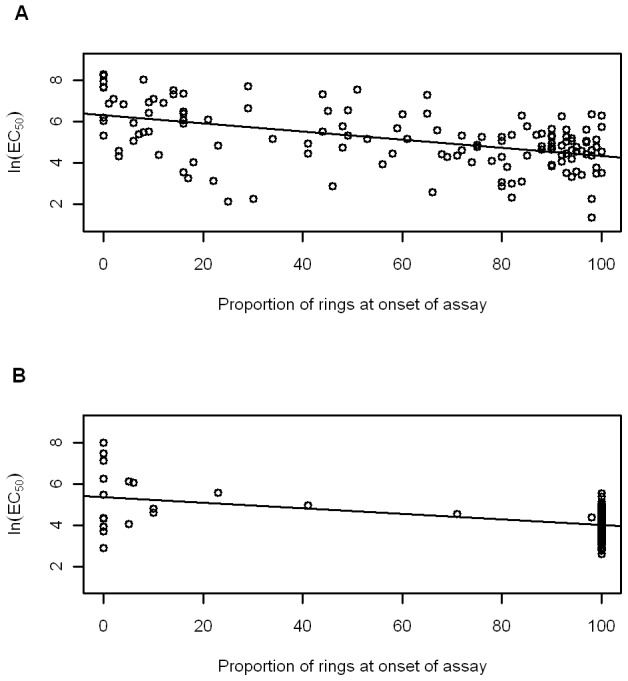
Relationship between the proportion of ring stage parasites at start of assay (0 hours) and ln(EC_50_). The figure depicts relationships for (A) *P. vivax* and (B) *P. falciparum*.

There was a significant negative association between the CQ assay duration (hours) and the ln (EC_50_) values for *P. vivax* (*r^2^* = 0.219, *p*<0.001; Figure 1a). A similar, but weaker relationship was observed for *P. falciparum* (*r^2^* = 0.097, *p*<0.001; Figure 1b). Figure 1 suggests some bimodality in the distribution of results, but this is predominantly due to the lack of sampling between ∼32 and 40 hours. The proportion of rings at the start of the assay was also significantly negatively associated with the ln(EC_50_) values for both *P. vivax* (*r^2^* = 0.245, *p*<0.001) and *P. falciparum* isolates (*r^2^* = 0.206, *p*<0.001; Figure 2). In both species there was a significant negative correlation between the proportion of rings at the onset of assay and assay duration (*r^2^* = 0.621, *p*<0.001; *r^2^* = 0.185, *p*<0.001, for *P. vivax* and *P. falciparum*, respectively).

**Figure 3:**
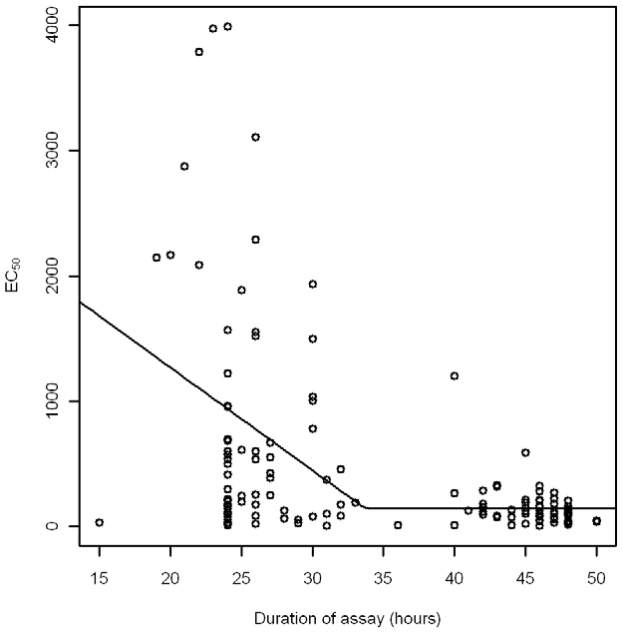
Threshold modelling results for the relationship between assay duration and estimated CQ EC_50_ values in *P. vivax*.

The threshold model for assay duration was successfully fit to the *P. vivax* data (Figure 3, Table 1). The threshold value, *c*, represents the point at which the assay duration was no longer significantly associated with estimates of EC_50_. We interpret this threshold as the point at which assay duration was sufficiently long to guarantee that the target parasite stage/s were present and exposed to the drug. Although there was a relationship between assay duration and EC_50_ for *P. falciparum* we were unable to fit a threshold model as fitting procedures did not identify an appropriate non-linear model and associated threshold point.

**Table 1:** Parameter estimates for threshold modelling of the relationship between assay duration and CQ EC_50_ in *P. vivax*.

Parameter	Estimate	95% Confidence Intervals		P-value
		Lower	Upper	
Threshold (*c*, in hours)	33.73	28.15	39.31	<0.001
Change in EC**_50_** per increase of one hour assay duration when assay duration<*c* (*a*)	−82.01	−131.69	−32.33	0.002
Mean EC**_50_** when duration>*c* (*b*, in nmols/l)	141.91	−19.05	302.87	0.086

**Figure 4:**
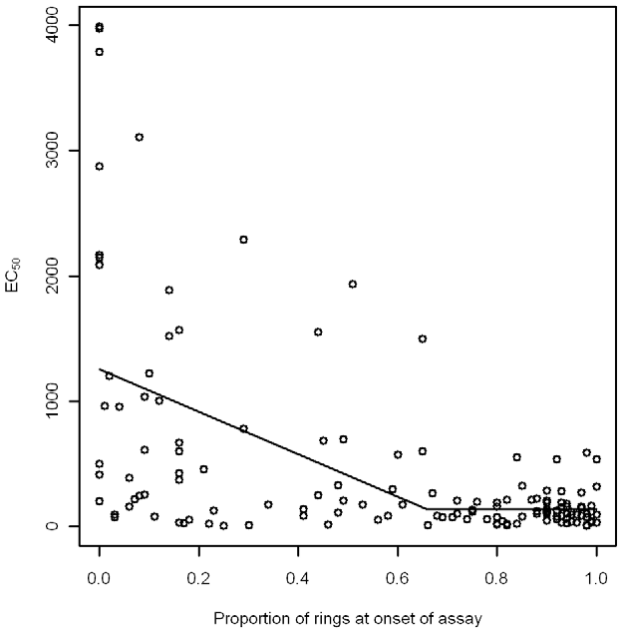
Threshold modelling results for the relationship between the proportion of rings in the initial sample and estimated CQ EC_50_ values in *P. vivax*.

A threshold model to determine the relationship between the initial proportion of rings and the EC_50_ was also only possible for *P. vivax*; we were unable to fit a threshold model to the *P. falciparum* data. For *P. vivax* the proportion of rings at the onset of assay was found to have a significant non-linear relationship with EC_50_ (Figure 4; Table 2). Samples with less than 65% ring stage parasites at time 0 had higher and more variable EC_50_ values than those isolates with a greater proportion of ring stage parasites.

**Table 2:** Parameter estimates for threshold modelling of the relationship between the proportion of rings samples at hour 0 and CQ EC_50_ in *P. vivax*.

Parameter	Estimate	95% Confidence Intervals		P-value
		Lower	Upper	
Threshold (*c*)	0.65	0.42	0.89	<0.001
Change in EC**_50_** when proportion of rings at hour 0<*c* (*a*)	−1693.2	−2470.7	−9 15.7	<0.001
Mean EC**_50_** when proportion of rings at hour 0>*c* (*b*, in nmols/l)	139.69	−7.49	286.87	0.065

### Comparisons with other drugs

Of the six other drugs tested, threshold models were successfully fitted to data for amodiaquine and mefloquine in *P. vivax*. Confidence intervals around the estimated threshold points showed distinct overlap, suggesting no significant differences between the two drugs, or CQ (Table 3).

**Table 3:** Comparison of the threshold points for amodiaquine, chloroquine and mefloquine, when analysing the relationship between assay duration and EC_50_ in *P. vivax*.

Drug	Threshold Estimate (*c*, hrs)	95% Confidence Intervals		P-value
		Lower	Upper	
Chloroquine	33.73	28.15	39.31	<0.001
Amodiaquine	31.13	27.84	35.7	0.01
Mefloquine	39.14	32.94	45.34	<0.001

### Characterising the duration of the erythrocytic cycle

In the simulations of the growth dynamics under *ex vivo* assay conditions, the λ parameter for the Poisson distributions represented the duration of each parasite life cycle stage in *P. vivax*. The mean λ was 19.1 hours (95% CI 11, 29) for rings, 23.1 hours (95% CI 11, 36) for trophozoites, and 2.2 hours (95% CI 0, 7) for the schizonts. The total duration of the *P. vivax* erythrocytic cycle was estimated as 44.5 hours (95% CI 29, 62). Similar simulations of *P. falciparum* growth gave mean estimates of the λ parameter of 23.3 hours (95% CI 14, 33) for ring stage parasites, 21.4 hours (95% CI 11, 33) for trophozoites, and 3.7 hours (95% CI 0, 10) for schizonts; estimates produce a total duration of 48.4 hours (95% CI 32, 66). These estimates of an *ex vivo* life cycle do not include the age of the rings before the start of the assay and the growth time of schizonts after the end of the assay, thus the true life cycle is expected to be longer than reported.

To validate the methodology, the growth dynamics of a small number of SMT results were also simulated for *P. malariae* (n = 39) and *P. ovale* (n = 13) isolates. The total duration of the erythrocytic cycle was estimated to be 75.6 hours (95% CI 59, 93) in *P. malariae* and 53.9 hours (95% CI 34, 79) in *P. ovale*.

## Discussion

Despite being developed initially for *P. falciparum* parasites, the Schizont Maturation Test (SMT) has seen considerable use in testing drug susceptibility of *P. vivax*. However, little consideration has previously been given to whether the application of the SMT to *P. vivax* is appropriate. In this study, we demonstrate that the stage-specific dynamics of drug activity may impact on the validity of SMT results. If a particular drug targets ring stage parasites, but is tested in an isolate predominantly containing mature stage parasites, the drug will appear to be ineffective, resulting in an overestimate of resistance. Assay duration and the proportion of rings in the initial sample both provide proxy indicators of the likelihood that early stage parasites will be exposed to drugs. Previous experimental work has identified that trophozoite stage *P. vivax* parasites are insensitive to CQ [15], [16]; our results support this finding. We apply statistical analysis techniques to show that the heterogeneity of erythrocytic life cycle stages present in peripheral blood samples taken from patients infected with *P. vivax* necessitates additional criteria be applied to the SMT to ensure the validity of the results.

The significant negative relationship between the time of venepuncture and start of the SMT and duration of the SMT for *P. falciparum* was not unexpected. The onset of fever is usually associated with the start of a new erythrocytic cycle, meaning the sum of the time elapsed between establishment of the SMT and SMT duration will be more indicative of total duration of the erythrocytic cycle than the SMT duration alone. Hence, parasites which have a delay between venepuncture and start of SMT will enter the SMT in a more advanced state, requiring less time to develop to 40% schizonts. This effect is most likely more dominant in *P. falciparum* samples due to the high synchronicity in parasites when obtained from the patient, compared to *P. vivax*.

For both *P. vivax* and *P. falciparum* isolates, there were significant negative relationships between the duration of the assay and estimated EC_50_ values (i.e., short duration assays were associated with reduced susceptibility to drugs). There are a number of possible explanations for these results, none of which is mutually exclusive and all of which are specific to the drug/parasite combination:

1. Later stage parasites are more tolerant to the drug then ring stage parasites; the higher the proportion of ring stage parasites the lower the EC_50_,
2. The drug effect is cumulative over time which results in longer exposure times having a greater relative effect for the same drug concentration,
3. Resistant parasites grow faster.

When stage-specific drug activity is a consideration, as with CQ against *P. vivax* [15], [16], we hypothesised that samples more advanced in their development, as characterised by short assay duration or a low proportion of ring stage parasites in the initial blood sample, would appear to be less susceptible to a drug because a significant number of parasites present in the assay had developed beyond the target stage. Support for this hypothesis is provided by the threshold modelling. While the linear models we have described are appropriate for the *P. falciparum* SMTs, examination of the trends in *P. vivax* suggest a distinctly non-linear pattern for some drugs, including CQ. The threshold modelling indicates that the EC_50_ for CQ in *P. vivax* parasites stabilises once the sample has been exposed to the drug for at least 33.7 hours (CI 28.2, 39.3). The simulation modelling of parasite development time suggests that *P. vivax* parasites spend an average of 25.3 hours as trophozoites and schizonts before termination of the assay. If the SMT is terminated when the control well reaches 40% schizonts, and this occurs after 33.7 hours (mean duration threshold), it follows that the SMT must have exposed sufficient ring stage parasites to the drug, resulting in EC_50_ reaching its minimum. Combining the results from the threshold and life cycle modelling, an assay lasting 33.7 hours will have exposed 86.5% of the parasites to CQ for at least one hour at ring stage. Using the upper 95% confidence limit for mean assay duration, a more conservative threshold estimate of 39 hours, we can predict that 95% of the parasites were exposed to CQ for at least 2 hours at ring stage. We propose that this threshold represents the duration of the assay which is sufficient to guarantee that a significant proportion of the ring stage *P. vivax* parasites have been exposed to the drug (i.e. there is no longer any association between duration and EC_50_). Assay durations shorter than this threshold expose a greater proportion of tolerant mature stage parasites to the drug, and thus the EC_50_ values derived from these samples will be artificially elevated.

Similar dynamics are apparent when examining the composition of the initial blood samples. The disparity in the degree of developmental stage heterogeneity in the initial samples between *P. vivax* and *P. falciparum* is striking. *P. falciparum* isolates are markedly more synchronous, presumably because the mature stages (trophozoites and schizonts) are sequestered in deep tissues and organs, rather than in the peripheral circulation. Nearly all *P. falciparum* samples contain only rings, and are, therefore, ideal for SMT. In contrast, *P. vivax* isolates tend to show significantly greater heterogeneity in the life cycle stages, with trophozoites and schizonts regularly occurring. The presence of these advanced stages at the onset of SMT makes the interpretation of drug susceptibility results more difficult. Our modelling suggests that the initial sample should contain a minimum of 66% ring stage parasites, preferably >90% ring stage parasites (upper 95% confidence interval of the threshold parameter), to ensure the target life cycle stages are sufficiently present in a sample. However, this significantly reduces the number of samples from which drug sensitivity data can be obtained potentially introducing a sampling bias. It should be noted that, by selecting only those samples that achieve 40% schizonts, we also introduce bias against those parasites that do not grow well in culture.

Processes for synchronising parasitemia have also been proposed as a means of decreasing stage heterogeneity of *Plasmodium* isolates for *ex vivo* characterisation [20], [21]. Although such methods may have utility and permit testing of some field isolates that would otherwise be excluded from testing, the removal of mature trophozoite parasites inevitably results in a reduction in parasite count, which is itself another important parameter for reliable quantification of parasite growth.

An alternate approach to specifying a definitive threshold is to apply the same types of threshold models which we have developed here using all available data. Such an approach would have three advantages. First, it would allow all the field samples to be used, thus reducing the potential for bias in the SMT samples. Second, it would allow the development of resistance to be monitored over time by looking for changes in the threshold duration and minimum EC_50_. Third, it can be used to look for stage-specific drug action in current and new antimalarial drugs. Similar patterns and threshold values were found for CQ, amodiaquine and mefloquine, suggesting all of these drugs have their main effect on ring stage *P. vivax* parasites. Such a relationship was not observed for the other antimalarials investigated (i.e., artesunate, lumefantrine, piperaquine and pyronaridine). Differences between the stage specific activity of each drug and its variation between parasite species may prove highly informative in elucidating the mechanisms of drug action as well as innate and acquired drug resistance. While it is always possible to investigate stage-specific drug activity using carefully planned laboratory experiments, as reported by Russell et al. [15], the methodology presented here can identify stage specificity through far less laborious means, and can use previously collated results.

Our simulation of parasite development in the SMT and subsequent estimate for the duration of each life cycle stage is the first attempt to model parasite development times for *P. vivax*. It is important to note that the estimates are relative to the parasite development in the restricted conditions of the SMT control well and may not represent the length of the life stages *in vivo*, or indeed in potential *in vitro* culture. It should also be expected that the estimated duration of the ring stage underestimates the true duration due to the delay in obtaining the blood sample after parasite rupture and establishing the SMT. More expansive sampling over the first 24 hours of the assay would likely reduce the confidence intervals of the estimated development times.

In summary, a threshold modelling approach was applied to data from a modified SMT to investigate resistance to CQ in *P. vivax*. We identified patterns which suggest a non-linear relationship between drug susceptibility in the parasite and both the duration of an assay and the proportion of ring stage parasites in the initial sample, which signifies tolerance of late stage parasites to CQ. Consequently, we recommend that *P. vivax* isolates should contain a minimum of 66% ring stage life cycle stages, and that assay duration should exceed 34 hours to ensure this stage-specific effect does not artificially inflate the reported EC_50_. More conservative thresholds would require a minimum of 90% ring stage parasites and a minimum assay duration of 40 hours. An alternative approach would be to use the statistical methodology which has been developed. For field researchers, this threshold modelling approach will allow for increased confidence in the reliability of resistance results. This approach also provides a novel means of detecting stage-specific drug activity for new antimalarials, as demonstrated by our analysis of the susceptibility to amodiaquine and mefloquine.
